# Diagnostic Accuracy of Antigen ELISA and Western Blot IgG for Neurocysticercosis in People Living with HIV/AIDS in Tanzania

**DOI:** 10.3390/tropicalmed10090246

**Published:** 2025-08-29

**Authors:** Yakobo Lema, Ulrich Fabien Prodjinotho, Charles Makasi, Marrywinnie A. Nanyaro, Frank Asenga, Andrew Kilale, Sayoki Mfinanga, Charlotte Rüther, Andrea Sylvia Winkler, Eligius F. Lyamuya, Bernard J. Ngowi, Mkunde Chachage, Clarissa Prazeres da Costa

**Affiliations:** 1 Muhimbili Medical Research Center, National Institute for Medical Research (NIMR), Dar es Salaam P.O. Box 9653, Tanzania; charlesmakasi2021@gmail.com (C.M.); winnienanyaro@gmail.com (M.A.N.); frankassenga98@gmail.com (F.A.); kilale@yahoo.com (A.K.); gsmfinanga@yahoo.com (S.M.); b_ngowi@yahoo.co.uk (B.J.N.); 2 Institute for Medical Microbiology, Immunology, and Hygiene, Technical University of Munich (TUM), 80333 Munich, Germany; abien.prodjinotho@tum.de; 3 Center for Global Health, TUM School of Medicine and Health, Technical University of Munich (TUM), 80333 Munich, Germany; andrea.winkler@tum.de; 4 Institute of Public Health, Kilimanjaro Christian Medical University College, Moshi P.O. Box 2240, Tanzania; 5 Department of Neuroradiology, RoMed Hospital Rosenheim, 83022 Rosenheim, Germany; charlotte.ruether@web.de; 6 Department of Neurology, TUM University Hospital, Klinikum Rechts der Isar, Technical University Munich (TUM), 80333 Munich, Germany; 7 Department of Community Medicine and Global Health, Institute of Health and Society, University of Oslo, 0315 Oslo, Norway; 8 Department of Global Health and Social Medicine, Harvard Medical School, Boston, MA 02115, USA; 9 Department of Microbiology & Immunology, Muhimbili University of Health & Allied Sciences (MUHAS), Dar es Salaam P.O. Box 65001, Tanzania; eligius_lyamuya@yahoo.com; 10 Mbeya College of Health & Allied Sciences, University of Dar Es Salaam, Mbeya P.O. Box 608, Tanzania; mchachage@gmail.com; 11 Mbeya Medical Research Center, National Institute for Medical Research (NIMR), Mbeya P.O. Box 2410, Tanzania; 12 German Center for Infection Research (DZIF), Partner Site Munich, 80802 Munich, Germany

**Keywords:** neurocysticercosis, *Taenia solium*, HIV infections, enzyme-linked immunosorbent assay, western blotting, diagnostic accuracy, sensitivity, specificity, sub-Saharan Africa, resource-limited settings

## Abstract

**Background:** Neurocysticercosis (NCC) and HIV co-infection frequently occur in sub-Saharan Africa, yet the accuracy of available serological tests for NCC in immunosuppressed patients is uncertain. **Methodology:** We performed a cross-sectional diagnostic study on 101 people living with HIV from two endemic districts in Tanzania. Participants provided serum for cysticercosis antigen ELISA and Western Blot IgG; any positive result prompted neuroimaging investigation with cerebral computed tomography. NCC was diagnosed according to the 2017 revised Del Brutto criteria based on cCT according to Del Brutto criteria modified to exclude serology. Sensitivity, specificity, and area under the receiver–operating–characteristic curve (AUC) were calculated and adjusted for CD4 count and HIV stage. Two algorithms were compared: parallel testing (“either-test-positive”) and sequential screening (Ag ELISA screen, western blot IgG confirm). **Results:** NCC prevalence was 23%. Western Blot IgG outperformed Ag ELISA (sensitivity 57% vs. 30%; specificity 87% vs. 86%; AUC 0.73 vs. 0.57). Western blot IgG sensitivity declined to 54% when CD4 < 500 cells µL^−1^, while Ag ELISA remained low. Western blot IgG positivity independently predicted NCC (adjusted odds ratio 4.1, 95% CI 1.4–11.9); Ag ELISA did not. When we counted a positive if either test was positive (parallel rule), sensitivity rose to 78% and NPV to 87%. When we ran Ag ELISA only if IgG was negative (sequential rule), we saved 70% of IgG strips, kept specificity at 95%, and PPV at 69%, but sensitivity fell to 39%. **Conclusions:** Western blot IgG is the most reliable single serological test for NCC in PLHIV. Parallel testing increased sensitivity and NPV and may suit better primary-level facilities without routine imaging. Sequential testing achieved high specificity, PPV, and conserved test kits, making it ideal for centers with limited reagents or scanner access. Tiered use of these assays can streamline NCC diagnosis in *T. solium* endemic, resource-limited settings.

## 1. Introduction

Neurocysticercosis (NCC), a central nervous system parasitic infection caused by *Taenia solium* larvae, affects an estimated 2.5–8.3 million people globally [1]. Humans acquire the infection through the fecal-oral route by ingesting *T. solium* eggs, which develop into cysts in various tissues, most critically the brain. NCC is associated with an estimated burden of ~2.8 million disability-adjusted life years (DALYs) [2,3]. The highest NCC-related DALY rates occur in sub-Saharan Africa (SSA), whereby NCC accounts for a large proportion of adult-onset seizures (30–50% of late-onset epilepsy) [4,5]. In Tanzania, where the parasite is endemic, studies have shown that roughly one-third of adults with epilepsy have NCC lesions on brain imaging [6]. A systematic review of SSA reported that nearly one-fifth of people with epilepsy (PWE) had NCC, and meta-analysis reported a pooled NCC prevalence of ~22% among PWE in SSA, though with significant geographic variation (6–37%) [7]. Within Tanzania, NCC’s contribution to epilepsy also varies widely by region. For example, a community-based study in rural northern Tanzania (Haydom area) found that 17.5% of PWE had NCC on neuroimaging [8]. In contrast, in urban Dar es Salaam, only ~2% of PWE had NCC lesions [8]. This substantial burden of NCC-related epilepsy contributes to high morbidity and underscores the need for effective control measures. Untreated NCC can lead to acute epileptic seizures and potentially also epilepsy and/or other neurological disorders, such as acute and chronic headaches, and may even contribute to increased mortality in PWE, highlighting its impact on individuals and public health in general [1].

According to the Del Brutto diagnostic criteria, first proposed in 2001 and later revised in 2017, the clinical diagnosis of definitive NCC needs cCT or MRI [9]. Clinicians in SSA often treat epilepsy patients symptomatically or use serum assays that detect parasite antigens or antibodies, as the region has fewer than one CT scanner per million people [10,11,12].

According to the National AIDS Control Programme (NACP), Tanzania has an adult HIV prevalence of approximately 4.7%, which is roughly 1.4 million people living with HIV/AIDS [13]. The co-endemicity of HIV and NCC has important clinical implications as these infections overlap, control of epileptic seizures and epilepsy becomes more difficult, and can lead to poor health outcomes in affected communities [14,15]. However, evidence for the accuracy of serological tests in these coinfected patients is scarce. HIV infection leads to progressive loss of CD4^+^ T-cells and immune system dysfunction, including B-cell impairment, and may blunt antibody titers [16,17]. For antigen-based assays, different cyst stages secrete different antigens that could affect these tests differently [18,19]. Few studies, such as Noormahomed et al. (2014), who found Ag ELISA sensitivity fell from 72% at CD4 ≥ 500 cells/µL to 45% at CD4 < 200 cells/µL, Garcia et al. (2018), who reported Western Blot sensitivities of up to 100% for multiple viable cysts but reduced sensitivity (60–70%) for single viable or degenerating cysts, and highly variable sensitivity for calcified lesions, and Parija and Gireesh (2009), who observed that lowered immune response in HIV patients can reduce the sensitivity of antibody detection for cysticercosis, have stratified test performance by CD4 count or cyst stage, leaving clinicians in co-endemic resource-limited settings without clear guidance [16,20,21].

The primary aim was to determine the diagnostic accuracy of serological tests in PLHIV. We compared a circulating-antigen ELISA and a Western blot IgG against the cCT. We estimated sensitivity (Se), specificity (Sp), and area under the receiver–operating–characteristic curve (AUC). We examined how immune status and cyst viability modified these metrics to inform the development of practical, standardized diagnostic algorithms tailored to resource-constrained settings.

## 2. Methods

### 2.1. Study Design

This nested cross-sectional analysis was conducted from June 2018 to March 2021 in the Southern Highlands of Tanzania, specifically in the Chunya district (Mbeya region) and Iringa rural districts (Iringa region). In this study, we evaluated the diagnostic accuracy of cysticercosis antigen ELISA (Ag-ELISA) and Western Blot IgG in PLHIV, using the cCT as the neuroimaging-based reference standard.

Our study population comprised 101 PLHIV adults aged 18–65 years who were recruited from Care and Treatment Centres (CTCs) irrespective of neurological symptoms as part of a larger ongoing study. For this specific diagnostic accuracy analysis, we included PLHIV who had results for serological tests, Ag-ELISA, Western Blot IgG, and cCT.

### 2.2. Sample Collection and Laboratory Analysis

#### Blood Collection and Processing

We collected 15 mL of venous blood from each participant, transferring 5 mL into K_3_-EDTA tubes for HIV viral-load testing and 10 mL into serum-separator tubes for serology and immunoassay. Within 2 h, we centrifuged all tubes at 1500× *g* for 15 min. We saved serum aliquots for cysticercosis Western-blot IgG, antigen ELISA assays, and plasma aliquots for viral-load quantification. We then dispensed the aliquots into cryovials bearing anonymized study identifiers and stored them at −20 °C in monitored freezers until batch analysis.

### 2.3. Serological Testing for T. solium Cysticercosis (Index Tests)

#### 2.3.1. Antigen Detection

We detected circulating antigens of viable *T. solium* metacestodes with a commercial ELISA kit (Cysticercosis Ag ELISA, Advanced Practical Diagnostics BV, Turnhout, Belgium; ref 650501; sensitivity 100% for infections with ≥2 cysts; specificity 99.6%). We precipitated proteins with trichloroacetic acid, neutralized the supernatant, and incubated the treated samples in microtiter wells coated with monoclonal antibody B158C11A10. We then added peroxidase-conjugated monoclonal antibody B60H8A4, measuring absorbance at 450 nm, and called specimens positive when the antigen index was ≥1.3.

#### 2.3.2. Antibody Detection

We used the LDBio Cysticercosis Western Blot IgG kit (LDBio Diagnostics, lyon, France; lot WB202103; reported sensitivity 97.5% and specificity 100%) to detect *T. solium*-specific IgG. Following the manufacturer’s protocol, we electrophoretically separated porcine cysticercus antigens, transferred them to nitrocellulose strips, and incubated them with participant serum. We visualized bound antibodies as purple bands after adding alkaline-phosphatase–conjugated anti-human IgG. We deemed a specimen positive when at least two of five diagnostic glycoprotein bands (6–8, 12, 23–26, 39, or 50–55 kDa) appeared.

### 2.4. HIV Viral Load and CD4^+^ T-Cell Count

For this study, we used PLHIV, who had documented infection status in their CTC medical records. Plasma viral load was quantified using the Xpert^®^ HIV-1 Viral Load assay (Cepheid, Sunnyvale, CA, USA; lower detection limit: 40 copies/mL), and absolute CD4^+^ T-cell counts were determined using the BD FACSPresto™ System (BD Biosciences, San Jose, CA, USA) per the manufacturer’s instructions.

### 2.5. Neuroimaging Protocol and Analysis

We performed non-contrast and, when safe, contrast-enhanced cerebral CT on a GE Revolution 32-slice scanner (1.5-mm sections) at Mbeya Zonal Referral Hospital in all 101 patients, then transmitted all images to the Department of Neuroradiology at TUM University Hospital. An experienced neuroradiologist (CR), blinded to serology results, reviewed the scans in consultation with a neurologist (ASW) and classified lesions as active, inactive, or mixed using established imaging criteria. We then applied the 2017 Del Brutto criteria omitting all serologic components to avoid incorporation bias so that only CT findings (visible scolex, ring-enhancing or cystic lesions, calcifications) plus clinical and epidemiologic context formed our modified imaging-based reference standard for both case definition and stratified analyses.

### 2.6. NCC Case Definitions

We applied the 2017 Del Brutto criteria while deliberately omitting all serologic components to avoid incorporation bias. Our modified imaging-based reference standard included major imaging criteria—either a visible scolex or a cyst with ring enhancement—alongside minor imaging criteria of a single enhancing lesion or parenchymal calcification and epidemiologic criteria of documented exposure history or clinical seizures.

We defined Definitive NCC by unequivocal neuroimaging evidence of the parasite, such as a clearly visible scolex on CT, and Probable NCC by neuroimaging findings highly suggestive of infection—cystic lesions, enhancing lesions, or calcifications—in combination with supportive clinical or epidemiologic indicators. For diagnostic accuracy analyses, we grouped all participants meeting either Definitive or Probable criteria into a single “Neuroimaging Positive (NP)” category, and we labeled the remaining participants as “Neuroimaging Negative (NN)”.

### 2.7. Ethical Considerations

Ethical approval for this research was granted by the Muhimbili University of Health and Allied Sciences (MUHAS) Directorate of Research and Publications (Ref. DA.282/298/01.C), the National Health Research Ethics Committee of the National Institute for Medical Research (NIMR), Tanzania (Ref. NIMR/HQ/R.8a/Vol. IX/2529), and the Ethics Committee of the Technical University of Munich, School of Medicine and Health (Refs. 537/18 and 215/18S). All study procedures, including participant recruitment, sample collection, and data handling, adhered to strict national ethical guidelines provided by the NIMR and international standards such as the Declaration of Helsinki.

### 2.8. Statistical Analysis

We evaluated diagnostic accuracy by comparing cysticercosis Ag ELISA and Western Blot IgG results against the neuroimaging-based reference standard. We calculated Se, Sp, positive predictive value (PPV), negative predictive value (NPV), and likelihood ratios using standard formulas to quantify each test’s diagnostic performance. We then generated ROC curves and calculated the AUC for test discrimination between cysticercosis Ag ELISA and Western Blot IgG, using DeLong’s test for statistical significance. McNemar’s test assessed agreement between the paired diagnostic results of the two tests. We used multivariable logistic regression to examine the links between test positivity and clinical variables, including CD4 count and HIV stage, and to assess possible effect modification. To avoid sparse cells and improve clarity, we combined selected demographic categories—age, marital status, education, and occupation—before modeling. We then evaluated how immune status (CD4 counts) and cyst features affected diagnostic performance. The stratified analyses were considered exploratory to identify trends in diagnostic performance. Therefore, no formal correction for multiple comparisons was applied to these descriptive tables, as our primary inferential conclusions are based on the multivariable regression models. We conducted all analyses in Stata 17 (StataCorp LLC, College Station, TX, USA) (for the primary diagnostic accuracy analyses) and Python 2.6.1 within Jupyter Notebook (for development and evaluation of novel diagnostic algorithms), treating two-sided *p* < 0.05 as statistically significant.

### 2.9. Diagnostic Threshold Determination and Algorithm Development

For diagnostic tests, determining an “optimal cut-off” is crucial for classifying patients as positive or negative. This optimal point aims to maximize the test’s ability to correctly identify both those with the disease (sensitivity) and those without it (specificity). To achieve this, we employed ROC analysis and Youden’s Index.

We first generated ROC curves for Ag ELISA (considering its binary, optical density, and index formats) and Western Blot IgG, using the neuroimaging-based reference standard for all participants. An ROC curve graphically illustrates a test’s diagnostic accuracy across all possible cut-off points. For each curve, we calculated Youden’s Index, which is a measure that combines sensitivity and specificity (Sensitivity + Specificity − 1). The optimal diagnostic threshold for each test was then defined as the point on its ROC curve that yielded the maximum Youden’s Index.

Recognizing that immune status can influence test performance, we repeated this threshold determination procedure within two CD4 count strata: <500 and ≥500 cells µL^−1^. This allowed us to identify thresholds that might be more appropriate for different immune profiles. For Ag ELISA, semi-quantitative thresholds were estimated using participants with optical density or index data; where these data were not available, the binary Ag ELISA threshold was applied to ensure all participants were included in the analysis. While we also descriptively examined the influence of cyst stage (viability), the counts in each specific subgroup (e.g., active cysts within a particular CD4 stratum) were below ten, meaning they did not provide sufficient statistical power to quantitatively influence the optimal threshold selection for this algorithm.

Following the determination of these optimal thresholds, a decision-tree algorithm was constructed. The primary branching in this tree was based on the CD4 category (<500 vs. ≥500 cells µL^−1^,). No further branches based on cyst viability were created due to the limitations in sample size for each stratum. At the terminal nodes of this tree, two group rules were evaluated for classifying a participant as NCC-positive: (i) an “OR rule,” which classified a participant as NCC-positive if either Ag ELISA or Western Blot exceeded its respective optimal threshold for that CD4 stratum, and (ii) a “two-step (AND) rule,” in which Western Blot IgG was performed only on Ag ELISA positive specimens, and both tests had to be positive to confirm NCC.

The performance of these algorithms was rigorously assessed using ten-fold stratified cross-validation. For each fold, the ROC-derived thresholds were recalculated using the training data and then applied to the corresponding test fold. Sensitivity, specificity, positive predictive value (PPV), negative predictive value (NPV), and Area Under the Curve (AUC) were then computed. We report the mean and 95% confidence intervals for each metric across the ten folds to provide a robust estimate of algorithm performance.

## 3. Results

### 3.1. Demographic and Clinical Characteristics of Study Participants

This study included 101 PLHIV, 23 (22.8%) were neuroimaging positive (NP), and 78 (77.2%) were neuroimaging negative (NN). There were 64 males (63.4%) and 37 females (36.6%).

As shown in the Table 1 below age range for this study was 25–77 years old. The age distribution was similar between the two groups (NP vs. NN), with most participants in the 35–49 age group (49, 48.5%), followed by 34 (33.7%) in the 50 or older age group. Education levels were also comparable, with 40 (39.6%) having primary or lower education and 61 (60.4%) having secondary or higher education. Both NP and NN groups had fewer individuals with CD4 counts below 200 cells/µL (1 (4.3%) NP vs. 8 (10.3%) NN).

In the NP group, 20 participants (87.0%) had been on antiretroviral therapy (ART) for more than 24 months, compared to 53 (67.9%) in the NN group. Undetectable viral loads were observed in 20 (87.0%) NP and 69 (88.5%) NN participants. HIV/AIDS clinical staging showed a difference (*p* < 0.001), with no NP participants reported to be in WHO Stage IV compared to five (6.4%) in the NN group. A higher proportion of PLHIV in the NP group was in WHO Stage III (8, 34.8%) compared to the NN group (19, 24.4%).

### 3.2. Overall Diagnostic Accuracy of Ag ELISA and Western Blot IgG

The overall diagnostic performance of the serological assays is presented in Table 2. The Western Blot IgG was found to be the superior individual test for diagnosing NCC in this cohort. It demonstrated substantially higher sensitivity than the Ag-ELISA (57% vs. 30%) while maintaining a comparably high specificity (87% vs. 86%). This superior discriminatory power was confirmed by its significantly higher Area Under the Curve (AUC) of 0.73 compared to 0.57 for the Ag-ELISA (DeLong’s *p* = 0.02).

When the tests were combined in a parallel screening strategy (a positive result on either test), sensitivity increased markedly to 78%, though this came at the cost of reduced specificity (51%). This highlights the utility of this approach for ruling out disease, as reflected by its high negative predictive value of 87%. Direct comparison of the paired test results confirmed a significant difference in their diagnostic classification (McNemar’s test, *p* = 0.0005) and showed only slight agreement between them (κ = 0.11), indicating that the two assays often identify different patient populations.

**Figure 1:**
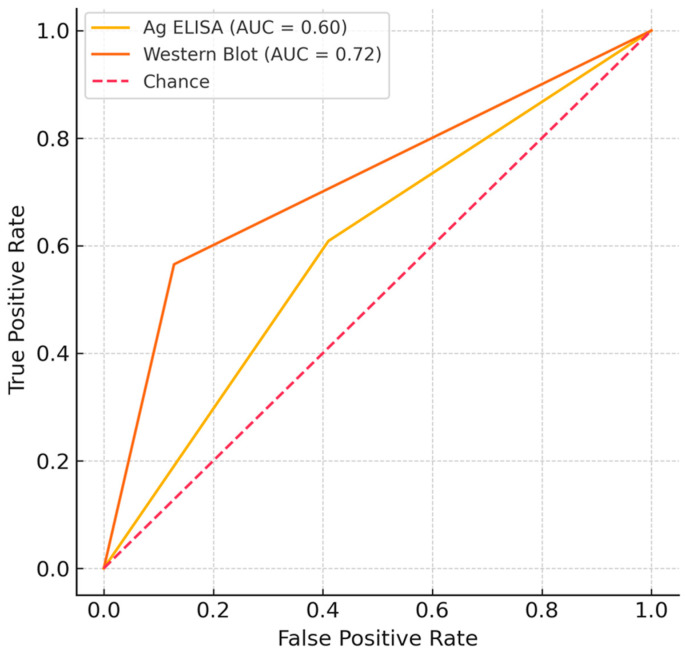
Receiver–operating–characteristic ROC curves for binary Ag ELISA and Western Blot. The diagonal dashed line represents a non-discriminatory test. AUC is Area Under the Curve.

The diagnostic ability of the cysticercosis Western Blot IgG was found to be superior to that of Ag ELISA in distinguishing neurocysticercosis cases. As visually represented in Figure 1, ROC analysis demonstrated a higher Area Under the Curve (AUC) for Western Blot IgG (AUC = 0.69, 95% CI: 0.62–0.77) compared to Ag ELISA (AUC = 0.58, 95% CI: 0.53–0.63). This difference in discriminatory power was statistically significant (DeLong’s *p* = 0.02).

### 3.3. Impact of Cyst Characteristics and CD4 Count on Diagnostic Performance

#### 3.3.1. Diagnostic Performance by Cyst Stage Stratification

We analyzed the two groups separately to compare test performance between active and inactive cyst stages. Table 3 shows that diagnostic performance varied with cyst viability. In patients with active cysts, the prevalence was 46% by cysticercosis Ag ELISA criteria and 23% by Western Blot IgG. Sensitivity was 20% (95% CI: 9–34%) for Ag ELISA and 35% (16–57%) for Western Blot, while specificity reached 98% (90–100%) and 97% (91–100%), respectively. Positive predictive values were 90% (56–100%) for Ag ELISA and 80% (44–97%) for Western Blot, and the corresponding negative predictive values were 59% (48–69%) and 83% (74–91%). In this subgroup, the positive likelihood ratios were 10.76 (1.42–81.81) for Ag ELISA and 13.57 (3.09–59.48) for Western Blot, with negative likelihood ratios of 0.82 (0.71–0.95) and 0.67 (0.50–0.90), respectively. Diagnostic odds ratios were 13.14 for Ag ELISA and 20.27 for Western Blot, with areas under the ROC curve of 0.59 (53–65%) and 0.66 (56–76%). For patients with inactive cysts, sensitivity fell to 11% (4–24%) for Ag ELISA and 22% (7–44%) for Western Blot, with specificity values of 85% (73–94%) and 90% (81–95%), respectively. In this group, both assays yielded a positive predictive value of 39% (14–68%), while the negative predictive values were 53% (42–64%) for Ag ELISA and 80% (70–87%) for Western Blot. The positive likelihood ratios were 0.75 (0.26–2.13) for Ag ELISA and 2.12 (0.77–5.85) for Western Blot, and the negative likelihood ratios were 1.04 (0.90–1.21) and 0.87 (0.69–1.10); diagnostic odds ratios were 0.72 (0.23–2.26) for Ag ELISA and 2.43 (0.75–8.01), with AUCs of 0.48 (42–55%) and 0.56 (46–65%), respectively.

Taken together, the diagnostic performance of both Ag ELISA and Western Blot IgG was significantly influenced by cyst viability, with a notable decrease in sensitivity observed for inactive cysts compared to active cysts.

#### 3.3.2. Diagnostic Performance Stratified by CD4 Count

Table 4 presents the diagnostic performance of the cysticercosis Ag ELISA and Western Blot IgG assays stratified by CD4 count categories (<500 and >500 cells µL^−1^). Due to the small numbers in the group <200 cells/µL (1 participant), we combined this group with the moderate immune-compromised group (200–500 cells µL^−1^) (12 participants) for the analysis. In participants with <500 cells µL^−1^, the prevalence was 40% for Ag ELISA and 21% for Western Blot. Sensitivity in this group was 28% (12–49%) for Ag ELISA and 54% (25–81%) for Western Blot, with specificities of 84% (69–94%) and 88% (76–95%), respectively. Positive predictive values were equivalent at 54% (25–81%) for both tests, whereas the negative predictive value was 64% (49–77%) for Ag ELISA compared with 88% (76–95%) for Western Blot. The positive likelihood ratio was 1.77 (0.67–4.66) for Ag ELISA and 4.49 (1.82–11.08) for Western Blot, and the negative likelihood ratio was 0.85 (0.65–1.13) versus 0.52 (0.29–0.95), with corresponding diagnostic odds ratios of 2.07 (0.63–6.86) and 8.56 (2.23–33.12) and AUCs of 0.56 (45–67%) and 0.71 (56–86%), respectively. Among participants with CD4 counts >500 cells µL^−1^ (N = 40, with 10 neuroimaging positive cases and 30 neuroimaging negative cases), the prevalence was 45% for Ag ELISA and 21% for Western Blot, with sensitivities of 33% (15–57%) and 60% (26–88%) and specificities of 89% (70–98%) and 89% (75–97%) for Ag ELISA and Western Blot, respectively. In this higher CD4 group, the positive predictive value was 70% (35–93%) for Ag ELISA compared with 60% (26–88%) for Western Blot, and the negative predictive values were 62% (45–78%) versus 89% (75–97%). Additionally, the positive likelihood ratios were 2.89 (0.85–9.83) for Ag ELISA and 5.55 (1.93–15.94) for Western Blot, with negative likelihood ratios of 0.75 (0.54–1.05) and 0.45 (0.21–0.97); diagnostic odds ratios were 3.83 (0.91–15.89) for Ag ELISA and 12.38 (2.56–60.66) for Western Blot, with AUCs of 0.61 (49–73%) and 0.75 (58–91%), respectively.

Overall, CD4 counts had a marked impact on assay sensitivity, particularly for Western Blot IgG, suggesting that immune status significantly modulates the ability of these serological tests to detect NCC.

#### 3.3.3. Diagnostic Performance Stratified by Duration on ART

We summarize the diagnostic results based on ART duration to determine if the duration of ART affects serological assay performance for NCC (Table 5). Among patients on ART for less than 24 months, the Ag ELISA (TsAg) had a prevalence of 44% (95% CI: 27–62%), with a sensitivity of 20% (4–48%) and a specificity of 90% (67–99%). Its positive predictive value (PPV) was 60% (15–95%) and its negative predictive value (NPV) was 59% (39–77%), yielding a positive likelihood ratio (LR+) of 1.9 (0.36–9.95), a negative likelihood ratio (LR−) of 0.89 (0.66–1.20), a diagnostic odds ratio (DOR) of 2.13 (0.36–12.36), and an area under the curve (AUC) of 0.55 (42–67%). In the same subgroup, the Western Blot IgG (TsAb) reported a prevalence of 15% (5–31%), a sensitivity of 40% (5–85%), and a specificity of 90% (73–98%). Its PPV and NPV were 40% (5–85%) and 90% (73–98%), respectively, with an LR+ of 3.87 (0.85–17.62), an LR− of 0.67 (0.32–1.38), a DOR of 5.78 (0.82–44.01), and an AUC of 0.65 (40–89%). For patients on ART for more than 24 months, the Ag ELISA showed a prevalence of 46% (34–59%), a sensitivity of 36% (19–55%), and a specificity of 81% (64–92%). Its PPV was 61% (36–83%), and its NPV was 59% (44–73%), with an LR+ of 1.82 (0.81–4.13), an LR− of 0.80 (0.59–1.09), a DOR of 2.28 (0.77–6.71), and an AUC of 0.58 (47–69%). In this group, the Western Blot IgG had a prevalence of 27% (17–39%), a sensitivity of 61% (36–83%), and a specificity of 86% (73–94%), with a PPV of 61% (36–83%) and an NPV of 86% (73–94%). Its LR+ was 4.28 (1.96–9.32), LR− was 0.45 (0.25–0.82), DOR was 9.43 (2.8–31.92), and AUC was 0.73 (61–86%). Comparative metrics revealed statistically significant differences between the paired test results in both ART duration groups, with McNemar’s χ^2^ values of 8.33 (df = 1, *p* = 0.004) and 5.45 (df = 1, *p* = 0.019) and Cohen’s κ values of 0.230 (64.71% agreement) for the <24-month group and 0.0415 (53.73% agreement) for the >24-month group.

In summary, the duration on antiretroviral therapy notably influenced serological test performance, with longer ART duration appearing to improve Western Blot IgG sensitivity, indicating a potential role for immune reconstitution in test outcomes.

### 3.4. Factors Affecting Diagnostic Performance (Regression Analysis)

Logistic regression analysis was performed to identify factors associated with diagnostic test positivity for cysticercosis among PLHIV (Table 6). In unadjusted logistic regression analyses, a positive result on the combined serological tests was associated with an odds ratio (OR) of 2.61 (95% CI: 1.12–6.07; *p* = 0.026) for NCC, while Western Blot positivity yielded an OR of 4.65 (1.77–12.26; *p* = 0.002) and Ag ELISA positivity an OR of 1.12 (0.49–2.57; *p* = 0.793). In adjusted analyses that accounted for CD4 count and HIV clinical stage, the OR for combined test positivity increased to 6.06 (1.33–27.7; *p* = 0.02) and for Western Blot positivity to 4.06 (1.39–11.92; *p* = 0.011). In contrast, Ag ELISA positivity in the adjusted model was associated with an OR of 0.24 (0.05–1.14; *p* = 0.074). For CD4 count, patients with >500 cells µL^−1^ had an unadjusted OR of 1.81 (0.79–4.16; *p* = 0.16) and an adjusted OR of 1.85 (0.72–4.75; *p* = 0.201) relative to those with <500 cells µL^−1^, while for HIV clinical stage, taking stage I as the reference, Stage II had an unadjusted OR of 0.85 (0.28–2.62; *p* = 0.777) and an adjusted OR of 0.89 (0.25–3.10; *p* = 0.851), Stage III had an unadjusted OR of 2.09 (0.80–5.42; *p* = 0.13) and an adjusted OR of 2.1 (0.73–6.07; *p* = 0.169), and Stage IV had an unadjusted OR of 1 (*p* = 1) with no adjusted estimate provided. Finally, when stratified by months on ART, patients on ART for more than 24 months showed an unadjusted OR of 1.50 (0.48–4.69; *p* = 0.482) and an adjusted OR of 2.11 (0.56–8.03; *p* = 0.272) compared to those on ART for less than 24 months. These findings suggest that, in our model, both immunologic status and duration on ART may influence the association between serological test positivity and NCC.

### 3.5. Diagnostic Algorithm Development

ROC analysis identified the following optimal cut-offs (Table 7; Supplementary Figure S1): binary Ag ELISA ratio ≥ 1, optical density ≥ 0.437, index ≥ 7.32, and Western Blot IgG positive when ≥2 diagnostic bands were present. Identical thresholds were observed after stratification by CD4 count (<500 vs. ≥500 cells µL^−1^).

A decision tree with a single CD4 split (<500, ≥500 cells µL^−1^) was applied. Two group rules were evaluated at the terminal nodes: (i) an OR rule (Ag ELISA positive or Western Blot IgG positive) and (ii) a two-step rule in which Western Blot IgG was performed only on Ag ELISA positive samples, and both tests had to be positive. Semi-quantitative Ag values replaced the binary ratio where available (76/101 participants); the binary ratio was used when semi-quantitative data were missing.

Ten-fold stratified cross-validation produced the diagnostic metrics summarized in Table 8. For the OR rule with binary Ag, the mean sensitivity was 0.78 (95% CI 0.64–0.93), specificity 0.51 (0.36–0.65), PPV 0.34 (0.28–0.40), NPV 0.87 (0.76–0.98), and AUC 0.64 (0.55–0.73). Incorporating semi-quantitative Ag values increased specificity to 0.71 and PPV to 0.41, while maintaining NPV at 0.89.

The two-step rule yielded specificity 0.95 (0.88–1.00), PPV 0.69 (0.51–0.87), sensitivity 0.39 (0.19–0.60), NPV 0.84 (0.74–0.94), and AUC 0.67 (0.55–0.78). Sensitivity was higher in participants with CD4 <500 cells µL^−1^ (0.84) than in those with CD4 ≥500 cells µL^−1^ (0.70), whereas specificity varied by less than one percentage point.

## 4. Discussion

This study evaluated the diagnostic accuracy of cysticercosis antigen ELISA and Western Blot IgG to diagnose NCC in PLHIV in Tanzania, using an imaging-based reference standard (2017 Del Brutto criteria modified to exclude serology). Our findings indicate that Western Blot IgG demonstrated superior overall discriminatory ability compared to Ag ELISA (AUC 0.73 vs. 0.57), thus showing a substantial relative gain in overall discriminatory power. We then tested whether test performance is influenced by immune status (CD4 count), clinical stage, time on antiretroviral therapy, or cyst characteristics. Lastly, we explored whether pairing these serological results with routine clinical data can deliver a practical diagnostic algorithm for clinics without ready cCT or MRI access.

Cysticercosis Western Blot IgG outperformed the antigen ELISA in detecting NCC in PLHIV. Western Blot achieved higher sensitivity than Ag ELISA, with both tests showing similar specificity. The moderate specificity observed for either test individually is biologically plausible. Antibodies may continue to exist even after the clearance of parasites, while antigens might indicate transient or extra-parenchymal infections [22]. Therefore, integrating both testing methods enhances detection capabilities; for instance, our parallel rule achieved a substantially higher sensitivity compared to either test alone, thereby decreasing the likelihood of missing NCC cases.

The overall sensitivity of Western Blot IgG and Ag ELISA against our imaging-based reference standard (Del Brutto criteria minus serology) in PLHIV is notably lower than often reported in studies of immunocompetent individuals, where Western Blot sensitivities typically range from 80–98% and Ag ELISA sensitivities for multiple viable cysts can approach 100% [23,24]. For Western Blot, persistent immune activation and dysregulation despite CD4 recovery can impair antibody production or avidity, reducing detection [25]. Although PLHIV more frequently present with active-stage cysts, which should increase antigen release, our participants had low-burden or calcified lesions that shed minimal antigen. These observations highlight the need to incorporate parasite dynamics, lesion viability, and assay performance when interpreting serologic and antigen-based diagnostics in PLHIV [24].

Test performance varied by both host CD4 count and cyst stage. Both assays detected a higher proportion of active NCC cases, whereas sensitivity for inactive (calcified) cysts fell below 25% for both tests. While these stratified results suggest performance is highly dependent on cyst viability, a formal statistical test for interaction was not performed due to the limited statistical power within these subgroups. This pattern aligns with *T. solium* biology; viable cysts secrete antigen detectable by Ag ELISA, while calcified lesions release minimal antigen and yield poor sensitivity [14,23]. Antibody responses detected by Western Blot are also typically stronger against active cysts, which present a broader range of antigens [26]. Our results, showing drastically reduced sensitivities for both tests in the presence of inactive (calcified) cysts, highlight the limitation of current serological assays in diagnosing calcified NCC even in PLHIV [27,28]. This reinforces the need for neuroimaging for definitive diagnosis, especially calcified lesions. When CD4 counts fell below 500 cells µL^−1^, Western Blot IgG sensitivity dropped to 54% while specificity remained unchanged. In contrast, the antigen ELISA remained low in sensitivity with little variation upon CD4 T cell decline. This aligns with a previous study in Mozambique reporting lower cysticercosis antibody rates in patients with lower CD4 counts [16]. Ag ELISA sensitivity remained low regardless of CD4 count, suggesting that factors beyond systemic immune cell counts, such as localized immune responses or the specific nature of antigen secretion by cysts, might be more critical for this assay in HIV co-infection.

Patients on antiretroviral therapy for more than 24 months showed higher Western Blot IgG sensitivity without loss of specificity, reinforcing the role of immune reconstitution in enhancing antibody detection. This finding parallels Parija et al.’s report from India, where restored immunity in HIV-positive patients reduced false negatives, underscoring the dynamic interplay between ART and the host response to *T. solium* [21].

Multivariable logistic regression confirmed that Western Blot IgG positivity is still usable as a stand-alone test of NCC in PLHIV. After adjusting for CD4 count and HIV clinical stage, patients positive on cysticercosis Western Blot IgG had four times higher odds of harboring cysts in the brain compared with Western Blot-negative peers. Classifying a participant as positive when “either” test was positive resulted in a sixfold increase in odds of being positive, showing that combined antigen and antibody detection captures additional true infections missed by either assay alone. After adjusting for immune status in the multivariable model, antigen ELISA positivity no longer reached statistical significance, indicating that its apparent association with NCC was driven by cyst viability or the degree of immunosuppression. This is in line with the manufacturer’s recommendation, which states that the antigen test only detects viable cysts and earlier reports of extra-parenchymal cysts being common with advanced HIV, and they tend to be less antigenic. These findings highlight cysticercosis Western Blot IgG and, even better, the running of both serological assays on the same sample and calling the patient positive if “either” the antigen ELISA or the Western Blot IgG is positive as the most reliable serological predictors of active NCC in these coinfected patients.

When Western Blot IgG and antigen ELISA are performed simultaneously under a parallel “OR rule” (a positive result on either assay defines a positive screen), the strategy’s high sensitivity (78%) and high negative predictive value (87%) make it a powerful tool for ruling out disease. This allows clinicians to confidently exclude NCC when both tests are negative. Although WHO guidelines do not endorse serologic assays as standalone screening tools in place of imaging, our results suggest that combining these assays could serve as a pragmatic interim strategy for HIV or epilepsy clinics without routine access to neuroimaging. In contrast, a sequential (two-step) pathway screens first with the less costly antigen ELISA and tests only ELISA-positive sera with Western Blot IgG, counting a case as positive only when both assays concur. This strategy cut Western Blot IgG reagent use by about 70% in our study, pushed specificity to 0.95, and lifted the positive predictive value to 0.69, though sensitivity fell to 0.39. The high specificity means that almost every patient referred for imaging truly harbors infection, an essential feature where scanner time and laboratory budgets are tightly rationed. Consequently, this algorithm suits district or regional hospitals that must confirm the disease before committing limited resources to advanced diagnostics or referral.

This study has some limitations that need to be considered. A primary limitation is the potential for verification bias, as the reference standard (cCT) was performed only in patients with positive serology rather than on the entire cohort. This design was necessitated by the ethical and logistical challenges of exposing all participants, many of whom were asymptomatic and serologically negative, to radiation and the high costs of imaging. This may have led to an overestimation of sensitivity and an underestimation of specificity, and our results should be interpreted with this in mind.

Additionally, the modest sample size of confirmed NCC cases limits the precision of subgroup estimates. Another limitation is that investigation with cCT may miss NCC lesions because of slice thickness (most CT scanners in low-income countries are older models with thicker slices), and sensitivity for extraparenchymal lesions is generally low on cCT. Therefore, cCT is an imperfect reference standard for NCC [29]. Lastly, the potential for cross-reactivity or non-NCC-related causes of positive test results, despite the high specificity of the Ag ELISA for viable *T. solium* cysts [30,31]. We cannot entirely rule out that some “false positives” were due to infections outside the central nervous system, such as muscles we did not scan.

Future research should aim to validate the proposed diagnostic algorithms in larger, prospective cohorts of PLHIV with suspected NCC across multiple sites to assess their generalizability and real-world performance. Clinical trials could measure whether screening HIV-positive individuals with new-onset seizures using Ag ELISA and IgG Blot (with confirmatory imaging for positives) leads to earlier NCC detection, improved seizure control, or more cost-effective use of CT scans compared to symptom-based referral alone. Such studies will inform guidelines on integrating NCC screening into routine HIV care in endemic areas.

## 5. Conclusions

Western Blot IgG provided better discrimination than the cysticercosis antigen ELISA for diagnosing NCC in PLHIV. Its diagnostic value remained robust after adjustment for CD4 count, HIV stage, and patient duration on ART, whereas the antigen assay lost significance once these factors were considered. A parallel (an “OR”) rule is well suited for primary health facilities that must rule out disease before referring patients for imaging services. A sequential algorithm in which we first screen with Ag-ELISA and perform Western Blot IgG only on Ag-positive samples maximizes specificity and conserves Western Blot reagents, making it attractive in settings where both laboratory capacity and imaging access are tightly rationed, although this is balanced by a very low sensitivity (39%) that would fail to detect the majority of cases. Together, these findings provide a strong rationale for the prospective evaluation of these tests in practical, tiered strategies for HIV clinics in *Taenia solium* endemic areas.

## Figures and Tables

**Table 1:** Demographic and Clinical Characteristics of PLHIV by Neuroimaging Results for NCC.

Variables	Total (101)	NP (23)	NN (78)	*p*-Value
**Age group (years)**				
<35	18 (17.8)	4 (17.4)	14 (18.0)	0.915
35–49	49 (48.5)	12 (52.2)	37 (47.4)	
≥50	34 (33.7)	7 (30.4)	27 (34.6)	
**Sex**				
Male	64 (63.4)	16 (69.6)	48 (61.5)	0.483
Female	37 (36.6)	7 (30.4)	30 (38.5)	
**Marital status**				
Married	6 (5.9)	0 (0.0)	6 (7.7)	0.157
Single/Unmarried ⁑	73 (72.3)	20 (87.0)	53 (67.9)	
Previous married	22 (21.8)	3 (13.0)	19 (24.4)	
**Education Level**				
Primary or below	40 (39.6)	8 (34.8)	32 (41.0)	0.591
Secondary or Higher	61 (60.4)	15 (65.2)	46 (59.0)	
**Occupation**				
Farmers	81 (80.2)	18 (78.3)	63 (80.8)	0.921
Formal Employment	15 (14.9)	4 (17.4)	11 (14.1)	
Others #	5 (4.9)	1 (4.3)	4 (5.1)	
**CD4+ T-Cell count (cells/µL)** ⁑				
<200	9 (8.9)	1 (4.3)	8 (10.3)	0.666
200–500	52 (51.5)	12 (52.2)	40 (51.3)	
>500	40 (39.6)	10 (43.5)	30 (38.5)	
**Months on ART**				
<6	22 (21.8)	3 (13.0)	19 (24.4)	0.157
6–24	6 (5.9)	0 (0.0)	6 (7.7)	
>24	73 (72.3)	20 (87.0)	53 (67.9)	
**HIV viral load (copies/mL)** ⁑				
Undetectable	89 (88.1)	20 (87.0)	69 (88.5)	0.136
<1000	3 (3.0)	2 (8.7)	1 (1.3)	
>1000	9 (8.9)	1 (4.3)	8 (10.3)	
**WHO HIV/AIDS Clinical Stage**				
Stage I	44 (43.1)	10 (43.5)	34 (43.6)	<0.001
Stage II	25 (24.5)	5 (21.7)	20 (25.6)	
Stage III	27 (26.5)	8 (34.8)	19 (24.4)	
Stage IV	5 (4.9)	0 (0.0)	5 (6.4)	

# is combined (unemployed, miners, and small businesses). NP is neuroimaging positive, and NN is neuroimaging negative. *p*-values indicate significance at *p* < 0.05 (Pearson’s chi-square test). ⁑ Percentages may not total 100% due to rounding

**Table 2:** Diagnostic Accuracy of Cysticercosis Ag ELISA and Western Blot IgG versus Neuroimaging (reference standard).

Statistic	Combined (Ag/Ab)	Western Blot IgG	Ag ELISA
Prevalence	0.46 (0.36–0.56)	0.23 (0.15–0.32)	0.46 (0.36–0.56)
Sensitivity	0.78 (0.64–0.93)	0.57 (0.35–0.77)	0.30 (0.18–0.46)
Specificity	0.51 (0.36–0.65)	0.87 (0.78–0.94)	0.86 (0.75–0.93)
PPV	0.34 (0.28–0.40)	0.57 (0.35–0.77)	0.61 (0.39–0.80)
NPV	0.87 (0.76–0.98)	0.87 (0.77–0.94)	0.59 (0.47–0.73)
LR+	1.59 (1.16–2.17)	4.41 (2.48–8.71)	1.86 (0.89–3.90)
LR−	0.43 (0.19–0.96)	0.49 (0.31–0.79)	0.83 (0.66–1.04)
DOR	3.70 (1.25–10.95)	8.84 (3.12–25.12)	2.24 (0.88–5.68)
AUC	0.65 (0.57–0.73)	0.73 (0.62–0.83)	0.57 (0.49–0.65)
**Comparison Metrics:**			
McNemar’s χ^2^, df, (*p*-value)	12.30, 1, (0.0005)		
Κ, Agreement Lvl	0.1051, 57.43%		

PPV is Positive Predictive Value, NPV is Negative Predictive Value, and AUC is Area Under the Curve. The combined test indicates a positive cysticercosis Ag ELISA or Western Blot IgG result. LR+ and LR− denote positive and negative likelihood ratios; AUC is calculated using DeLong’s test.

**Table 3:** Diagnostic Accuracy of Cysticercosis Ag ELISA and Western Blot IgG for NCC, Stratified by Cyst Stage.

Statistic	Active		Inactive	
	TsAg	TsAb	TsAg	TsAb
Prevalence	0.46 (0.36–0.56)	0.23 (0.15–0.32)	0.46 (0.36–0.56)	0.23 (0.15–0.32)
Sensitivity	0.20 (0.09–0.34)	0.35 (0.16–0.57)	0.11 (0.04–0.24)	0.22 (0.07–0.44)
Specificity	0.98 (0.90–1.00)	0.97 (0.91–1.00)	0.85 (0.73–0.94)	0.90 (0.81–0.95)
PPV	0.90 (0.56–1.00)	0.80 (0.44–0.97)	0.39 (0.14–0.68)	0.39 (0.14–0.68)
NPV	0.59 (0.48–0.69)	0.83 (0.74–0.91)	0.53 (0.42–0.64)	0.80 (0.70–0.87)
LR+	10.76 (1.42–81.81)	13.57 (3.09–59.48)	0.75 (0.26–2.13)	2.12 (0.77–5.85)
LR−	0.82 (0.71–0.95)	0.67 (0.50–0.9)	1.04 (0.9–1.21)	0.87 (0.69–1.1)
DOR	13.14 (2.32–74.28)	20.27 (6.99–58.73)	0.72 (0.23–2.26)	2.43 (0.75–8.01)
AUC	0.59 (0.53–0.65)	0.66 (0.56–0.76)	0.48 (0.42–0.55)	0.56 (0.46–0.65)
**Comparison Metrics:**				
McNemar’s χ^2^, df, (*p*-value)		0.33, 1, (0.564)		0.00, 1, (1.000)
Κappa, 95% CI, Agreement Lvl		−0.154, 70.0%		0.025, 53.85%

95% confidence intervals were computed using the Wilson score method.

**Table 4:** Diagnostic Accuracy of Cysticercosis Ag ELISA and Western Blot for NCC, Stratified by CD4^+^ T-Cell Count.

Statistic	CD4 Count < 500 Cells µL^−1^		CD4 Count > 500 Cells µL^−1^	
	TsAg	TsAb	TsAg	TsAb
Prevalence	0.40 (0.28–0.53)	0.21 (0.12–0.33)	0.45 (0.30–0.60)	0.21 (0.11–0.36)
Sensitivity	0.28 (0.12–0.49)	0.54 (0.25–0.81)	0.33 (0.15–0.57)	0.60 (0.26–0.88)
Specificity	0.84 (0.69–0.94)	0.88 (0.76–0.95)	0.89 (0.70–0.98)	0.89 (0.75–0.97)
PPV	0.54 (0.25–0.81)	0.54 (0.25–0.81)	0.70 (0.35–0.93)	0.60 (0.26–0.88)
NPV	0.64 (0.49–0.77)	0.88 (0.76–0.95)	0.62 (0.45–0.78)	0.89 (0.75–0.97)
LR+	1.77 (0.67, 4.66)	4.49 (1.82, 11.08)	2.89 (0.85, 9.83)	5.55 (1.93, 15.94)
LR−	0.85 (0.65, 1.13)	0.52 (0.29, 0.95)	0.75 (0.54, 1.05)	0.45 (0.21, 0.97)
DOR	2.07 (0.63, 6.86)	8.56 (2.23, 33.12)	3.83 (0.91, 15.89)	12.38 (2.56, 60.66)
AUC	0.56 (0.45, 0.67)	0.71 (0.56, 0.86)	0.61 (0.49, 0.73)	0.75 (0.58, 0.91)
**Comparison Metrics:**				
McNemar’s χ^2^, df, (*p*-value)		5.54, 1, (0.019)		7.12, 1, (0.008)
Κappa, 95% CI, Agreement Lvl		0.049, 57.38%		0.133, 57.50%

**Table 5:** Diagnostic Accuracy of Cysticercosis Ag ELISA and Western Blot for NCC, Stratified by Duration on ART.

Statistic	<24 Months on ART		>24 Months on ART	
	TsAg	TsAb	TsAg	TsAb
Prevalence	0.44 (0.27–0.62)	0.15 (0.05–0.31)	0.46 (0.34–0.59)	0.27 (0.17–0.39)
Sensitivity	0.2 (0.04–0.48)	0.4 (0.05–0.85)	0.36 (0.19–0.55)	0.61 (0.36–0.83)
Specificity	0.9 (0.67–0.99)	0.9 (0.73–0.98)	0.81 (0.64–0.92)	0.86 (0.73–0.94)
PPV	0.6 (0.15–0.95)	0.4 (0.05–0.85)	0.61 (0.36–0.83)	0.61 (0.36–0.83)
NPV	0.59 (0.39–0.77)	0.9 (0.73–0.98)	0.59 (0.44–0.73)	0.86 (0.73–0.94)
LR+	1.9 (0.36–9.95)	3.87 (0.85–17.62)	1.82 (0.81–4.13)	4.28 (1.96–9.32)
LR−	0.89 (0.66–1.2)	0.67 (0.32–1.38)	0.8 (0.59–1.09)	0.45 (0.25–0.82)
DOR	2.13 (0.36–12.36)	5.78 (0.82–44.01)	2.28 (0.77–6.71)	9.43 (2.8–31.92)
AUC	0.55 (0.42–0.67)	0.65 (0.4–0.89)	0.58 (0.47–0.69)	0.73 (0.61–0.86)
**Comparison Metrics:**				
McNemar’s χ^2^, df, (*p*-value)		8.33, 1, (0.004)		5.45, 1, (0.019)
Κappa, 95% CI, Agreement Lvl		0.230, 64.71%		0.0415, 53.73%

**Table 6:** Factors Associated with Diagnostic Test Positivity for NCC in PLHIV.

Factor	Category	cOR (95% CI)	*p*-Value	aOR (95% CI)	*p*-Value
Combined Pos	Pos vs. Neg	2.61 (1.12–6.07)	0.026	6.06 (1.33–27.7)	0.02
WB IgG		4.65 (1.77–12.26)	0.002	4.06 (1.39–11.92)	0.011
Ag Elisa		1.12 (0.49–2.57)	0.793	0.24 (0.05–1.14)	0.074
CD4 Cat	Ref: <500 cells µL^−1^				
>500 cells µL^−1^		1.81 (0.79–4.16)	0.16	1.85 (0.72–4.75)	0.201
HIV Stage	Ref: Stage I				
Stage II		0.85 (0.28–2.62)	0.777	0.89 (0.25–3.1)	0.851
Stage III		2.09 (0.8–5.42)	0.13	2.1 (0.73–6.07)	0.169
Stage IV		1		1	
ART Months	Ref: <24				
>24		2.13 (0.71–6.35)	0.175	2.11 (0.56–8.03)	0.272

**Table 7:** ROC-derived optimal cut-offs for Ag ELISA (binary and semi-quantitative) and Western Blot IgG, overall and by CD4 stratum.

CD4 Stratum	Test	Format	Optimal Cut-Off	Sens	Spec	AUC	*n*
All	Ag ELISA	Binary	≥1.0	0.54	0.63	0.58	101
All	Ag ELISA	O. D	≥0.437	0.53	0.87	0.67	76
All	Ag ELISA	Index	≥7.32	0.53	0.90	0.67	76
All	Western Blot	Binary	≥1.0	0.57	0.87	0.71	101
<500 cells µL^−1^	Ag ELISA	Binary	≥1.0	0.62	0.59	0.61	55
≥500 cells µL^−1^	Ag ELISA	Binary	≥1.0	0.47	0.67	0.57	46

**Table 8:** Cross-validated diagnostic performance of ensemble rules.

Algorithm	Ag Input	Sensitivity	Specificity	PPV	NPV	AUC
OR rule	Binary (ratio ≥ 1)	0.78 (0.64, 0.93)	0.51 (0.36, 0.65)	0.34 (0.28, 0.40)	0.87 (0.76, 0.98)	0.64 (0.55, 0.73)
OR rule	Semi-quantitative	0.70 ‡	0.71 ‡	0.41 ‡	0.89 ‡	0.70 ‡
Two-step rule (AND)	Binary (ratio ≥ 1 -> WB)	0.39 ‡	0.95 ‡	0.69 ‡	0.84 ‡	0.67 ‡

‡ Single-set estimates; confidence intervals not calculated because of missing OD values.

## Data Availability

The data presented in this study are available on request from the corresponding author. The data are not publicly available due to privacy and ethical restrictions.

## Supplementary Materials

The following supporting information can be downloaded at: https://www.mdpi.com/article/10.3390/tropicalmed10090246/s1. Figure S1: ROC Curve for all predictors in diagnostic algorithm development.
