# Supplementary figures and images for: Diagnostic Accuracy of Antigen ELISA and Western Blot IgG for Neurocysticercosis in People Living with HIV/AIDS in Tanzania
Source: Trop Med Infect Dis. 2025 Aug 29;10(9):246. doi: 10.3390/tropicalmed10090246 (PMC12474443; doi:10.3390/tropicalmed10090246)

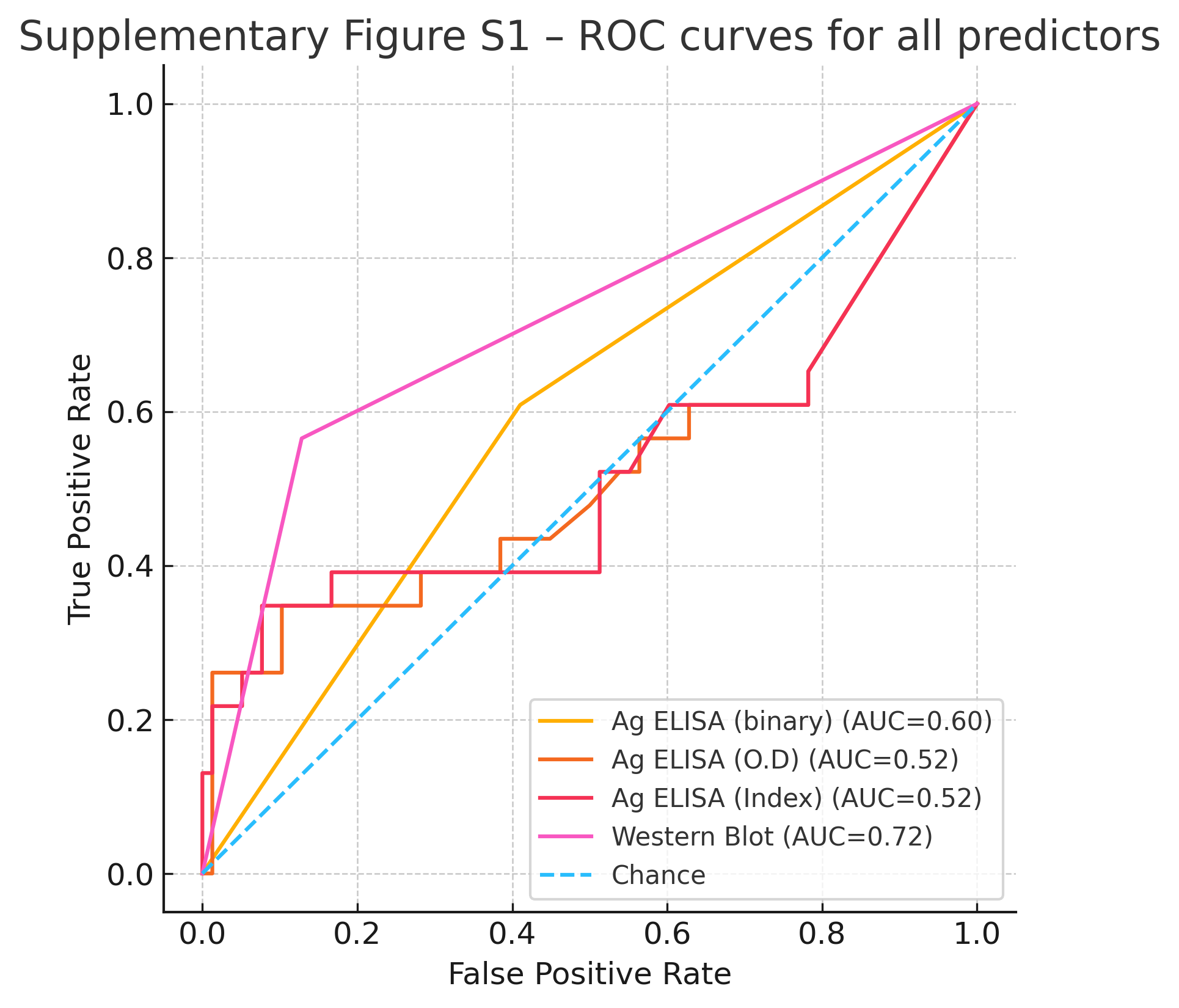

Supplement: Supplementary file 1 [file tropicalmed-10-00246-s001.zip › tropicalmed-3741731-supplementary.png]
